# Development and validation of the neighborhood environment walkability scale for youth across six continents

**DOI:** 10.1186/s12966-019-0890-6

**Published:** 2019-12-03

**Authors:** Ester Cerin, Terry L. Conway, Anthony Barnett, Melody Smith, Jenny Veitch, Kelli L. Cain, Ferdinand Salonna, Rodrigo S. Reis, Javier Molina-García, Erica Hinckson, Wan Abdul Manan Wan Muda, Ranjit Mohan Anjana, Delfien van Dyck, Adewale L. Oyeyemi, Anna Timperio, Lars Breum Christiansen, Josef Mitáš, Jorge Mota, Mika Moran, Mohammed Zakiul Islam, Robin R. Mellecker, James F. Sallis

**Affiliations:** 1 Mary MacKillop Institute for Health Research, Australian Catholic University, Melbourne, Australia; 2 School of Public Health, The University of Hong Kong, Hong Kong, China; 3 Department of Family Medicine and Public Health, University of California, San Diego, USA; 4 School of Nursing, The University of Auckland, Auckland, New Zealand; 5 Institute for Physical Activity and Nutrition (IPAN), Deakin University, Geelong, Australia; 6 Institute of Active Lifestyle, Palacky University of Olomouc, Olomouc, Czech Republic; 7 Prevention Research Center, Brown School, Washington University in St. Louis, St Louis, USA; 8 Department of Teaching of Corporal Expression, University of Valencia, Valencia, Spain; 9 School of Sport & Recreation, Auckland University of Technology, Auckland, New Zealand; 10 Khazanah Research Institute, Kuala Lumpur, Malaysia; 11 Madras Diabetes Research Foundation & Dr. Mohan’s Diabetes Specialties Centre, Chennai, India; 12 Department of Movement and Sport Sciences, Ghent University, Ghent, Belgium; 13 Department of Physiotherapy, University of Maiduguri, Maiduguri, Nigeria; 14 Department of Sports Science and Clinical Biomechanics, University of Southern Denmark, Odense, Denmark; 15 Research Centre in Physical Activity, Health and Leisure, University of Porto, Porto, Portugal; 16 Institute for Urban and Regional Development, University of California Berkeley, Berkeley, USA; 17 School of Public Health, University of Haifa, 3498839 Haifa, Israel; 18 Department of Architecture, Bangladesh University of Engineering & Technology, Dhaka, Bangladesh; 19 The University of Hong Kong, Hong Kong, China

**Keywords:** Built environment, Questionnaire, Confirmatory factor analysis, Pooled analyses, Global, Adolescents

## Abstract

**Background:**

The IPEN International Physical Activity and Environment Network Adolescent project was conducted using common study protocols to document the strength, shape, and generalizability of associations of perceived neighborhood environment attributes with adolescents’ physical activity and overweight/obesity using data from 15 countries. Countries did not use identical versions of the Neighborhood Environment Walkability Scale for Youth (NEWS-Y) to measure perceived neighborhood environment attributes. Therefore, this study derived a measurement model for NEWS-Y items common to all IPEN Adolescent countries and developed a scoring protocol for the IPEN Adolescent version of the NEWS-Y (NEWS-Y-IPEN) that maximizes between-country comparability of responses. Additionally, this study examined between- and within-country variability, and construct validity of the NEWS-Y-IPEN subscales in relation to neighborhood-level socio-economic status and walkability.

**Methods:**

Adolescents and one of their parents (*N* = 5714 dyads) were recruited from neighborhoods varying in walkability and socio-economic status. To measure perceived neighborhood environment, 14 countries administered the NEWS-Y to parents and one country to adolescents. Confirmatory factor analysis was used to derive comparable country-specific measurement models of the NEWS-Y-IPEN. Country-specific standard deviations quantified within-country variability in the NEWS-Y-IPEN subscales, while linear mixed models determined the percentage of subscale variance due to between-country differences. To examine the construct validity of NEWS-Y-IPEN subscales, we estimated their associations with the categorical measures of area-level walkability and socio-economic status.

**Results:**

Final country-specific measurement models of the factor-analyzable NEWS-Y-IPEN items provided acceptable levels of fit to the data and shared the same factorial structure with five latent factors (Accessibility and walking facilities; Traffic safety; Pedestrian infrastructure and safety; Safety from crime; and Aesthetics). All subscales showed sufficient levels of within-country variability. Residential density had the highest level of between-country variability. Associations between NEWS-Y-IPEN subscales and area-level walkability and socio-economic status provided strong evidence of construct validity.

**Conclusions:**

A robust measurement model and common scoring protocol of NEWS-Y for the IPEN Adolescent project (NEWS-Y-IPEN) were derived. The NEWS-Y-IPEN possesses good factorial and construct validity, and is able to capture between-country variability in perceived neighborhood environments. Future studies employing NEWS-Y-IPEN should use the proposed scoring protocol to facilitate cross-study comparisons and interpretation of findings.

## Background

In the last decade, findings from systematic reviews have supported the importance of the neighborhood environment for engagement in physical activity (PA) across the lifespan [1–5]. It is particularly imperative to understand how attributes of the neighborhood environment influence PA in adolescents as they have lower levels of behavioral autonomy than adults and, hence, are more likely to be affected by the local environment [2]. Internationally, the majority of adolescents fail to meet PA guidelines [6] and exhibit marked decreases in PA as they transition from childhood to adulthood [7]. Thus, adolescents are a key target population for PA promotion.

Studies focusing on adolescents have consistently reported positive associations of overall PA with neighborhood PA infrastructure and equipment and null associations with residential density and environmental aesthetics [8]. The evidence about other neighborhood attributes, including street connectivity, pedestrian infrastructure, access to services and facilities, traffic safety and safety from crime, has been inconclusive due to the small number of studies or divergent findings [8, 9]. The presence of inconsistent associations across studies could be due to genuine differences in effects across geographical regions and cultures, differences in methods (e.g., measures of the neighborhood environment and PA) or restricted variability in environmental exposures [10]. All published studies in this field have originated from single cities or countries and used a variety of measures. It is therefore unclear whether the potential effects of specific environmental features on adolescents’ PA are universal or country-specific. This information is necessary to inform evidence-based global and national interventions to increase or prevent decreases in PA in this demographic group [11]. The International Physical Activity and the Environment Network (IPEN) [12] was established to address this knowledge gap by stimulating multi-country research on environmental correlates of PA in various age groups, including adolescents, using comparable study designs and measures.

The IPEN Adolescent study was conducted using common study designs, protocols, and measures in 15 countries across six continents to document the strength, shape, and generalizability of associations of attributes of the neighborhood environment with adolescents’ PA and overweight/obesity. By collecting data from substantially diverse geographical locations, IPEN Adolescent maximized the variability in environmental exposures, health behaviors and health outcomes, allowing a more robust and accurate estimation of dose-response relationships than single-site studies. The study employed a modified version of the Neighborhood Environment Walkability Scale for Youth (NEWS-Y) [13] to measure perceived neighborhood attributes hypothesized to influence PA in adolescents - such as, residential density, proximity of recreation facilities, walking/cycling facilities, and aesthetics. The original NEWS-Y was derived from the NEWS for adults [14, 15], and was adapted for youth based on qualitative studies with youth and parents, then validated in US adolescents and parents [13]. NEWS-Y subscales showed acceptable test-retest reliability and construct validity (i.e., associations with PA), which tended to be higher for responses from parents than adolescents. Safety-related subscales of the NEWS-Y displayed good psychometric properties in Hong Kong Chinese adolescents [16]. However, the factorial validity of the NEWS-Y has never been examined. Thus, it is not known whether the a priori defined measurement model of the NEWS-Y is valid and applicable to various geographical locations, cultures and languages. This is an important consideration given that IPEN Adolescent aims to conduct pooled analyses of data from 15 countries across the globe, requiring between-site comparability of measures. Evidence for the factorial validity is also an important consideration for other studies that used or are planning to use the NEWS-Y.

Other psychometric characteristics of the NEWS-Y worth investigating are convergent and divergent validity, which are two aspects of construct validity [17]. Convergent validity examines whether constructs that are expected to be related are, in fact, related, while divergent validity establishes whether constructs hypothesized not to be related are, in fact, unrelated. In the context of this study, tests of convergent and divergent validity could be based on the associations of specific perceived neighborhood attributes, as measured by their relevant NEWS-Y subscales, with objectively-assessed neighborhood walkability and socio-economic status (SES). Although perceptions of the environment are sometimes weakly related to the objective environment, the former are influenced by the latter [18]. For example, a recent study using IPEN Adult data from 10 countries found strong positive associations between objective measures of the neighborhood built environment and their perceived counterparts assessed using the NEWS for adults [19]. In the context of the present study, we would expect NEWS-Y subscales measuring perceived attributes of the environment corresponding or related to the components of a walkability index (dwelling density, street intersection density and land use mix) [20] to be significantly associated with the walkability index. These would, for example, include perceived dwelling density, street connectivity, access to services and land use mix – diversity. We would expect perceived access to recreational facilities to be unrelated to objective neighborhood walkability as defined above. We would also expect perceived dwelling density, street connectivity and access to services to be unrelated to objective neighborhood SES given that the IPEN study sampling strategy requires recruitment of participants from high and low walkable communities balanced by neighborhood SES [21].

In summary, as findings on the associations between neighborhood environment features and adolescents’ PA are either scarce or inconsistent, and mostly derived from single-site studies with limited environmental variability, IPEN Adolescent aims to address these knowledge gaps by analyzing comparable pooled data from 15 geographically and culturally diverse countries. To ensure data comparability, it is important to validate and harmonize the exposure and outcome measures across study sites. One of these measures is the NEWS-Y.

Mirroring the methods and procedures used in the multi-country validation of the NEWS employed in the IPEN Adult study [10], the main aims of the present paper were to 1) identify subsets of comparable NEWS-Y items used across IPEN Adolescent countries, derive a measurement model for these items that would be appropriate for all sites, and develop a scoring protocol for the IPEN Adolescent version of the NEWS-Y (NEWS-Y-IPEN) that maximizes between-site comparability of responses; 2) report on the between- and within-country variability in the NEWS-Y-IPEN subscales; and 3) examine construct validity of the NEWS-Y-IPEN subscales in relation to objective aspects of the neighborhood environment (i.e., area-level measures of high and low SES and walkability).

## Methods

### Neighborhood selection

IPEN Adolescent data were collected in 18 cities/regions from 15 countries across six continents (Table 1). Area-level stratification was used to maximize within-site variability in environmental exposures deemed to influence PA. Specifically, adolescents and one of their parents were recruited from schools and/or residential areas located in neighborhoods stratified by SES and walkability into high SES/high walkable, high SES/low walkable, low SES/high walkable and low SES/low walkable [22–26].

**Table 1:** Overall and country-specific sample characteristics

Countries	All countries	Australia	Bangladesh	Belgium	Brazil	Czech Republic	Denmark	Hong Kong	India	Israel	Malaysia	New Zealand*	Nigeria	Portugal	Spain	USA
**Cities**		Melbourne	Dhaka	Ghent	Curitiba	Olomouc; Hradec Králové	Odense	Hong Kong	Chennai	Haifa	Kuala Lumpur & oth.	Auckland; Wellington	Gombe	Various cities	Valencia	Baltimore, Maryland; Seattle, Washington
Overall N	5714	238	86	279	491	183	197	1291	315	109	348	400	258	132	465	922
Adolescents’ age (years)																
Mean	14.4	14.5	13.9	13.4	14.1	14.2	13.0	14.3	13.8	15.5	14.3	14.6	15.3	16.0	16.5	14.1
SD	1.7	1.5	1.8	1.4	1.6	1.5	1.2	1.7	1.6	1.5	1.2	1.4	1.7	1.3	0.8	2.4
Adolescents’ sex																
Male, %	46.7	40.3	51.2	41.9	49.1	42.6	57.9	42.8	52.4	38.5	40.2	55.5	55.0	34.8	44.9	49.7
Highest education in household (%):																
Did not complete high school	15.0	10.5	4.7	17.6	19.1	11.5	10.7	18.4	34.6	7.3	24.1	22.5	6.2	30.3	10.8	0.8
High school graduate or some college	35.7	24.4	34.9	39.4	39.9	45.4	21.3	46.3	48.6	28.4	34.2	28.3	38.4	25.8	33.5	23.9
College graduate or more	49.3	65.1	60.5	43.1	40.9	43.2	68.0	35.2	16.8	64.2	41.7	49.3	55.4	43.9	55.7	75.4
Area-level SES																
High, %	48.2	52.1	46.5	52.3	43.4	41.0	54.3	46.5	48.3	41.3	49.4	47.8	39.9	56.1	53.5	50.1
Area-level walkability																
High, %	50.8	55.5	46.5	59.9	51.7	39.3	49.2	49.2	51.4	49.5	70.1	50.7	56.6	50.8	37.4	49.6

*Notes.* N = participants (adolescent and parent dyads) with information on adolescent’s age, sex, school, administrative area of residence, area-level socio-economic status, highest educational attainment in household, and NEWS-Y data from parents; *SES* = socio-economic status; ^a^ New Zealand collected NEWS-Y data from adolescents only

#### Area-level SES

Census data on median household or personal income were used to determine *area-level SES* in Australia, Belgium, Brazil, Denmark, Hong Kong SAR (China), New Zealand and the USA. Malaysia used self-reported income aggregated by administrative units, which were then split at the median into low or high categories. Bangladesh, Portugal and Spain defined area-level SES using census data on education, while the Czech Republic and Israel employed census-based composite measures of area-level SES. Nigeria’s National Population Commission categorized the study-city’s enumeration units into low or high SES categories. India did not have SES-related census data for enumeration wards in their cities, so they relied on expert judgments of investigators (e.g., property values, aesthetics, building quality) to classify wards into low or high SES categories.

#### Area-level walkability

Area-level walkability was based on a walkability index constructed using geographic information systems (GIS), defined as a composite measure of residential density, intersection density and land use mix, with or without retail floor area ratio, in all countries except for Malaysia, India and Nigeria [20]. The Czech Republic, Denmark and USA included retail floor area ratio in their walkability index. Malaysia used a composite measure of residential and intersection density. Nigeria and India did not have GIS data, so they categorized enumeration units/wards as low- or high-walkable based on judgments by investigators and local land use experts familiar with walkability components. For example, high walkable neighborhoods in Gombe, Nigeria were characterized by high residential density, high concentration of non-residential land uses (retail shops, local markets and places of worship) and streets with short block length with many alternative routes to destinations. Low-walkable neighborhoods in Gombe, Nigeria were characterized by low residential density (predominantly separate, single family homes), few non-residential land uses, and streets with longer block length with fewer alternative routes to destinations.

The majority of countries with area-level SES and walkability measures used region-specific median values to classify administrative areas into low versus high groups for each dimension. However, several countries used more stringent criteria for determining groups, such as by creating deciles of SES and walkability values and then excluding recruitment from administrative areas in the middle deciles [27].

### Participants, recruitment and data collection

Brazil, Israel and the USA recruited participants (adolescents and one of their parents; *aka* adolescent-parent dyad) directly from residential addresses located in neighborhoods varying in SES and walkability. Belgium and India targeted both residential addresses and schools from such neighborhoods. Hong Kong SAR (China) recruited adolescent-parent dyads from schools situated in areas stratified by SES and walkability, and who resided in pre-selected administrative areas representing the four neighborhood types. The remaining nine countries selected participants from schools situated in areas stratified by SES and walkability, irrespective of the participants’ residential address. In doing so, they also attempted to obtain a balanced number of participants by type of residential neighborhood. New Zealand recruited adolescents only (no parents), with parents only asked to answer a few socio-demographic and neighborhood self-selection questions upon providing consent for their child to participate in the study. The age range for adolescent participants’ recruitment was 11 to 19 years.

Participants were contacted in person in all countries except the USA (mail and phone). Data collection was conducted from 2009 to 2016 globally, with an average data collection period within countries of 13.8 months. Surveys were self-administered (paper-and-pencil or online) in Australia, Belgium, Denmark, Hong Kong SAR, Israel, and the USA, and interviewer-administered in Bangladesh, Brazil, India, Malaysia, New Zealand, Nigeria, Portugal, and Spain. The Czech Republic used a combination of interviews and online self-completed surveys. Response rates ranged from 11.0 to 89.7%, with Bangladesh and Israel being unable to provide this information. All study sites obtained approval to conduct the study by the ethics committees of their local institutions. Written parental consents and adolescent assents were obtained prior to data collection.

The parent-version version of the NEWS-Y-IPEN was administered to parents of adolescents in 14 out of 15 countries. New Zealand was the only country administering the youth-version of the NEWS-Y-IPEN to adolescents. Thus, only data provided by adolescents was analyzed for New Zealand. The IPEN Adolescent coordinating center decided to focus on parental rather than adolescents’ perceptions of environmental attributes deemed to influence adolescents’ PA because previous studies have indicated that parents’ responses are more reliable than those provided by adolescents [13]. In addition, as parents largely determine their adolescent’s level of independent mobility [28], it is appropriate to assess their perceptions of the neighborhood environment. Prior analyses demonstrated acceptable associations (intraclass correlations) between parent and adolescent reports for most subscales [13]. Only participants with basic socio-demographic data and information on the administrative unit of residence and adolescent’s school were included in the analyses to enable adjustment for school-level and/or neighborhood-level clustering [29].

The sample included 5714 eligible participants, with country-specific sample sizes ranging from 86 (Bangladesh) to 1291 (Hong Kong SAR) (Table 1). The average age of the adolescents enrolled across countries ranged from 13.1 to 16.5 years. Adolescents’ sex was relatively balanced across country-specific samples, with the exception of Israel and Portugal where the percentage of males was considerably lower (< 40%). Participants’ residential area-level SES and walkability categories were relatively balanced in most countries. The Czech and Spanish samples had a disproportionately small proportion of participants from walkable neighborhoods (< 40%), while the opposite was observed for the Malaysian sample. In Australia, Bangladesh, Denmark, Israel and the USA, a large proportion of participants (> 60%) came from households with tertiary educational attainment.

### Measures

#### Neighborhood environment walkability scale for youth for the IPEN adolescent study (NEWS-Y-IPEN)

The original version of NEWS-Y was developed by Rosenberg and colleagues [13] to measure aspects of the neighborhood environment that may impact physical activity among adolescents and children. It has a parent and a youth version and consists of 67 items grouped into eight subscales measuring Residential density, Land use mix – diversity, Recreational facilities, Land use mix - access, Pedestrian and automobile traffic safety, Crime safety, Aesthetics, Walking/cycling facilities, and Street connectivity (Additional file 1: Table S1). For IPEN Adolescent, the coordinating center modified the NEWS-Y to create the NEWS-Y-IPEN as detailed in Additional file 1: Table S1. Non-English versions of the NEWS-Y-IPEN were forward-translated into the local language, back-translated into English and certified by the IPEN Adolescent coordinating center.

The original *Residential density* subscale included four items on the types of homes in one’s neighborhood (e.g., detached one-family houses to multi-family apartment buildings). Each item was rated on a 5-point scale to indicate how common each housing type was in the neighborhood (1 = none; 2 = a few; 3 = some; 4 = most; 5 = all). To capture more accurately between-country variability and a broader range in residential density, two housing-type items were added to the NEWS-Y-IPEN subscale corresponding to those used in the NEWS for the IPEN Adult study [10]. Mirroring the scoring used in IPEN Adult, responses on this subscale ranged from 0 (none) to 4 (all). Denmark did not include the item representing the highest level of residential density in their survey due to the lack of > 20-story high-rise residential buildings in their study site (Odense). The weights multiplied by the responses to the items of this subscale were based on the estimated number of units for each type of residential building and corresponded to those used in the IPEN Adult version of the NEWS scale (weights of 1 for item 1, single-family residences; 11 for item 2, multi-family houses of 1–3 stories; 25 for item 3, multi-family houses of 1–3 stories; 50 for item 4, multi-family houses of 7–12 stories; 75 for item 5, multi-family houses of 13–20; and 100 for item 6, multi-family houses of over 20 stories) [10]. A total residential density score was computed by summing all weighted items’ responses (Additional file 2: Table S2).

The *Land use mix – diversity* subscale of the original NEWS-Y consisted of items gauging perceived walking proximity from home to 20 types of destinations, 13 of which were included in the NEWS-Y-IPEN (Additional file 1: Table S1). Items measuring proximity to a hardware store, clothing store, video store, bookstore, fruit/vegetable market, hairdressers/barber shop and offices/worksites were omitted to reduce length and/or because they were not deemed to be highly relevant to adolescents. The original *Recreational facilities* subscale assessed perceived walking proximity from home to 14 types of recreational facilities. Nine of these facilities were included in NEWS-Y-IPEN (Additional file 1: Table S1). Recreational destinations more relevant to children or adults than adolescents were excluded (e.g., playgrounds). Responses ranged from 1 to 5 min walking distance (with a score of 5) to > 30-min walking distance (with a score of 1). Summary scores of land use mix – diversity (13 categories) and recreational facilities (9 categories) were computed by averaging ratings across the respective destinations.

The *Land use mix – access* subscale of the original NEWS-Y includes six items. Only two of these items (Difficult to find parking; Hilly streets) were retained in the NEWS-Y-IPEN (Additional file 1: Table S1). The other four were omitted to shorten the instrument given that they measure accessibility to services similar to those included in the Land use mix – diversity subscale. The original *Pedestrian and automobile traffic safety* and *Crime safety* subscales encompassed, respectively, seven and six items. To shorten the NEWS-Y-IPEN, an item deemed to be less representative of the construct was dropped from each subscale. The *Aesthetics* (four items) and *Walking/cycling facilities* (three items) subscales of the NEWS-Y-IPEN corresponded to those of the original NEWS-Y, while the modified *Street connectivity* subscale included only two of the three items from the original NEWS-Y (Additional file 1: Table S1). All the above items were rated on a 4-point Likert scale (1 = strongly disagree; 4 = strongly agree). Summary scores for each subscale were computed by averaging scores on the corresponding items (reverse scored when needed in the direction consistent with higher walkability and safety).

#### Socio-demographic characteristics

For the purpose of this paper, the following parent-reported socio-demographic characteristics were considered: child’s sex and age and highest educational attainment in the household.

### Data analyses

#### Country-specific measurement models of the NEWS-Y-IPEN and subscale scoring

Items measuring Residential density, Land use mix – diversity and Recreational facilities were not factor-analyzed because their subscales are not considered to represent unidimensional constructs. For example, recreational facilities such as parks, basketball courts and lakes do not necessarily co-occur. The same applies to buildings of different height (Residential density items).

Individual-level country-specific measurement models of the factor-analyzable items of the NEWS-Y-IPEN were obtained by conducting separate Confirmatory Factor Analyses (CFAs) for each country with a sufficiently large sample size (> 200 participants [30]). These were Australia, Belgium, Brazil, Hong Kong SAR (China), India, Malaysia, New Zealand, Nigeria, Spain and the USA, which had complete data on the NEWS-Y-IPEN and school-level and/or neighborhood-level identifiers (used to adjust for clustering in the data). For countries with a sufficient number of participants per school and/or administrative unit of recruitment (i.e., two or more), CFAs were conducted on within-area variance/covariance matrices representing estimates of individual-level relationships between items [31]. These were Brazil, Hong Kong SAR (China), Malaysia, Nigeria, Spain and USA. For the remaining countries, CFAs were conducted on raw data. Maximum Likelihood Estimation was used for all CFAs. A priori country-specific measurement models of the NEWS-Y were determined based on the available items across countries (Additional file 1: Table S1) and previous CFAs of the NEWS for adults [10, 14, 32]. The a priori measurement model of the NEWS-Y-IPEN included the following inter-correlated latent factors:

1. *Land use mix – access,* with two common items across all countries
2. *Pedestrian and automobile traffic safety,* with six common items
3. *Safety from crime,* with five common items
4. *Aesthetics,* with four common items
5. *Walking / cycling facilities,* with three common items
6. *Street connectivity,* with two common items

Models were re-specified using Jöreskog and Sörbom’s iterative model-generating approach [33]. The procedure included an inspection of standardized factor loadings, residual covariances, univariate Langrage multiplier tests, Wald tests and multivariate outliers, and was informed by theoretical considerations. We used a combination of model-fit indices recommended by Hu and Bentler [34] and Kline [35] to assess the goodness-of-fit of the measurement models. These included the Comparative Fit Index (CFI), the Standardized Root Mean Squared residual (SRMS) and the Root Mean Square Error of Approximation (RMSEA). Values of CFI ≥ 0.95, SRMS≤0.08 and RMSEA≤0.06 are supportive of good model fit. Because the CFI is sensitive to the magnitude of correlations between items [35], and these correlations are often modest for co-occurring environmental attributes [10], CFI values ≥0.90 were considered as indicative of good model fit if the RMSEA and SRMS met Hu and Bentler’s criteria [34]. We also reported the values for the χ^2^ test. CFAs were conducted using EQS. 6.3 (Multivariate Software Inc.; http:www.mvsoft.com/faq.htm).

#### Between- and within-country variability in the NEWS-Y-IPEN subscales

Country-specific means, and standard deviations were computed for each subscale of the final, common measurement model of the NEWS-Y-IPEN. For each subscale, we also computed the percentage of variance due to between country differences. This was estimated using empty (i.e., with no predictors) linear mixed models with random intercept at the administrative unit and country levels.

#### Construct validity of the NEWS-Y-IPEN

To examine the construct (convergent and divergent) validity of NEWS-Y-IPEN subscales, we examined their associations with the categorical (dichotomous) measures of area-level walkability and SES. These associations were estimated using generalized linear mixed models adjusted for country and accounting for clustering at the school and administrative unit levels. We hypothesized that subscales measuring characteristics such as residential density and availability/access to destinations would be positively related with area-level walkability (i.e., their scores would be higher in high- than low-walkable areas) because these characteristics are components of the walkability index used to select study areas [36]. We did not expect significant associations between these subscales and area-level SES because high and low walkable areas were balanced by SES as a result of the study design. We also hypothesized that neighborhood aesthetics and safety aspects would be positively related to area-level SES (i.e., their scores would be higher in high-SES areas) since higher-SES neighborhoods tend to have more aesthetically-pleasing buildings, and lower levels of crime and traffic [36–38]. Finally, based on findings from several studies [e.g., 37, 39–41], availability of different recreational facilities was expected to be better in high- than low-SES neighborhoods. Here, it is worth noting that the Recreation facilities subscale of the NEWS-Y-IPEN represents more a measure of availability (number of different facilities) rather than access (distance to the nearest facility). Had this subscale represented access to recreation facilities, we would have expected a negative association with area-level SES in line with many studies [e.g., 42–44]. All models were adjusted by child’s age and sex. Sensitivity analyses were conducted to examine the impact of basing area-level walkability and SES on expert opinion – namely, analyses were performed on the whole sample as well on a subsample that excluded countries that used expert opinion to classify areas by SES and walkability (Nigeria, Malaysia and India).

## Results

### Country-specific measurement models of NEWS-Y-IPEN and subscale scoring

CFAs were conducted only using data from the ten countries with a sufficient number of eligible participants. The a priori measurement model of the NEWS-Y-IPEN did not show an acceptable level of fit to the data of any country (Table 2). Specifically, RMSEA values were higher than 0.06 in all countries except for Brazil, indicating inadequate fit according to Hu and Bentler’s criteria [34]. Although the a priori measurement model for Brazil had acceptable values for RMSEA and SRMS, the associated CFI value was too low (< 0.90).

**Table 2:** Goodness-of-fit indices for a priori and re-specified country-specific measurement models of the NEWS-Y-IPEN

Country	A priori measurement models				Final measurement models			
	χ^2^ (df)	CFI	RMSEA (95% CI)	SRMR	χ^2^ (df)	CFI	RMSEA (95% CI)	SRMR
Australia	478 (194)	0.864	0.082 (0.073, 0.091)	0.078	192 (124)	0.958	0.050 (0.036, 0.064)	0.068
Belgium	612 (194)	0.860	0.094 (0.085, 0.102)	0.100	204 (125)	0.961	0.052 (0.039, 0.064)	0.078
Brazil	442 (194)	0.844	0.051 (0.045, 0.057)	0.056	209 (126)	0.935	0.037 (0.028, 0.045)	0.047
Hong Kong SAR	1268 (194)	0.905	0.066 (0.062, 0.069)	0.067	624 (128)	0.944	0.055 (0.051, 0.059)	0.056
India	441 (194)	0.869	0.066 (0.057, 0.073)	0.065	180 (129)	0.964	0.037 (0.023, 0.049)	0.061
Malaysia	493 (194)	0.889	0.068 (0.061, 0.076)	0.084	239 (125)	0.947	0.053 (0.042, 0.063)	0.070
New Zealand^a^	423 (194)	0.877	0.065 (0.058, 0.073)	0.072	240 (128)	0.914	0.048 (0.038, 0.057)	0.057
Nigeria	432 (194)	0.870	0.071 (0.062, 0.080)	0.078	211 (133)	0.951	0.049 (0.036, 0.061)	0.070
Spain	527 (194)	0.899	0.069 (0.063, 0.075)	0.064	308 (126)	0.948	0.056 (0.048, 0.064)	0.049
USA	933 (194)	0.897	0.064 (0.060, 0.068)	0.067	368 (129)	0.955	0.045 (0.039, 0.050)	0.065

*Notes.* All χ^2^ significant at the <.001 level, NEWS-Y-IPEN = Neighborhood Environment Walkability Scale for Youth for the IPEN Adolescent study, *df* degrees of freedom, *CFI* comparative fit index, *RMSEA* root mean square error of approximation, *SRMR* standardized root mean squared residuals, 95% *CI* 95% confidence intervals. ^a^New Zealand collected NEWS-Y data from adolescents only. Thus, for New Zealand, confirmatory factor analyses were performed on adolescent data, while for the other countries they were performed using parents’ data

An examination of the standardized factor loadings, standardized residuals, and Wald tests indicated several issues contributing to the misfit of the model to the data that were common to most countries. First, the item ‘Parking is difficult in shopping areas’ did not significantly load on the factor it was supposed to measure (Land use mix – access) for Australia, Belgium, Brazil, India, Hong Kong SAR and Malaysia, and/or displayed significantly larger loading on factors conceptually unrelated to ‘access to parking’ (e.g., in Belgium this item loaded on the latent factor Safety from crime, and in Australia on the latent factors Street connectivity and Walking / cycling facilities). Given the above, and the fact that most adolescents do not drive a car, this item was omitted from subsequent NEWS-Y-IPEN measurement models. Second, the item ‘Presence of grass / dirt between the streets and the sidewalks’ loaded on Aesthetics rather than Walking / cycling facilities in four countries (Hong Kong SAR, India, Nigeria and Spain) and did not significantly load on any of the latent factors in the Belgian sample. Third, rather than Aesthetics, the item ‘Presence of trees along the streets’ was related to the latent factor Walking / cycling facilities in the Malaysian, Hong Kong SAR, Spanish and USA samples, and to Land use mix – Access in Nigeria, and the item was unrelated to all factors in the Brazilian sample. Fourth, the item ‘High crime rate’ loaded more strongly on Pedestrian and automobile traffic safety than Safety from crime in the Australian, Malaysian and USA samples. It also had higher loadings on three to four latent factors other than Safety from crime in the samples from Hong Kong SAR and Nigeria, and it did not significantly load on any of the specified factors in the Brazilian sample. To ensure cross-country comparability in the structure of the NEWS-Y-IPEN measurement models, these four items were excluded from subsequent CFAs.

As standardized residuals, Wald tests and inter-factor correlations indicated the remaining items gauging Land use mix – access (‘Hilly streets make it difficult for me/my child to walk in’), Street connectivity (‘Less cul-de-sacs in neighborhood’ and ‘Many different routes for getting from place to place in our neighborhood’) and Walking / cycling facilities (‘Presence of sidewalks on most of the streets’ and ‘Sidewalks separated from the road / traffic by parked cars’) were consistently inter-correlated across all countries, they were made to load on a single latent factor named Accessibility and walking facilities. The latent factor Pedestrian and automobile traffic safety with six items was split into two 3-item correlated latent factors because the two sets of items were only weakly correlated in seven out of 10 countries. One of these new latent factors was named Traffic safety and included the items ‘Difficult / unpleasant for my child to walk due to traffic in the neighborhood’, ‘Speed of traffic usually slow (30 mph)’ and ‘Drivers drive faster than speed limit’. The other factor was named Pedestrian infrastructure and safety. It encompassed the items ‘Good lighting at night’, ‘Easy view of walkers / bikers from houses’ and ‘Cross-walks and signals to cross busy streets’. Apart from the above-mentioned modifications, all models were re-specified by allowing item error terms within latent factors to be correlated and constraining inter-factor correlations to zero when appropriate.

Table 2 shows that all re-specified final measurement models of the NEWS-Y-IPEN fitted the data sufficiently well (CFI ≥ 0.90, SRMS≤0.08 and RMSEA≤0.06). The final models for Australia, Belgium, India, Nigeria and the USA met the more stringent goodness-of-fit criteria proposed by Hu and Bentler [34]. Standardized factor loadings were statistically significant at a probability level of 0.001 and in the expected direction (Table 3). Most items’ standardized loadings had an absolute value greater than 0.30, indicating a significant relationship between the items and the factor they were supposed to measure [10, 45]. The final measurement models of NEWS-Y-IPEN were very similar across countries, with five latent factors, some of which were inter-correlated (Table 3). Latent factors with consistently high standardized loadings were Aesthetics and Safety from crime. Relatively low, albeit significant, standardized loadings on the Accessibility and walking facilities latent factor were observed for the items ‘Hilly streets make it difficult to walk in the neighborhood’ and ‘Less cul-de-sacs in the neighborhood’. The only measurement model based on adolescent-reported ratings of the NEWS-Y-IPEN (New Zealand) tended to show lower standardized item loadings on the first two latent factors (Accessibility and walking facilities; Traffic safety) than measurement models based on parent-reported ratings of the NEWS-Y-IPEN (all other countries). The average inter-factor correlations were low. In five of ten countries, Accessibility and walking facilities and Pedestrian infrastructure and safety were the latent factors with the strongest moderate-to-high inter-correlation (Table 3).

**Table 3:** Final country-specific measurement models of the NEWS-Y-IPEN

	Country									
	Australia	Belgium	Brazil	Hong Kong SAR	India	Malaysia	New Zealand*	Nigeria	Spain	USA
Factors and items	Standardized factor loadings									
Accessibility and walking facilities (AW)										
AW1. Hilly streets make it difficult to walk in the neighborhood	− 0.38	− 0.35	− 0.31	− 0.31	− 0.28	−0.31	− 0.25	− 0.29	− 0.34	− 0.34
AW2. Less cul-de-sacs in the neighborhood	0.29	0.36	0.37	0.36	0.31	0.39	0.24	0.33	0.39	0.32
AW3. Many different routes for getting from place to place in our neighborhood	0.52	0.48	0.26	0.53	0.33	0.36	0.32	0.42	0.40	0.41
AW4. Presence of sidewalks on most of the streets	0.46	0.92	0.54	0.57	0.84	0.78	0.53	0.71	0.70	0.89
AW5. Sidewalks separated from the road / traffic by parked cars	0.56	0.70	0.36	0.30	0.76	0.50	0.42	0.72	0.53	0.64
Traffic safety (TS)										
TS1. Difficult/unpleasant to walk due to traffic in the neighborhood	−0.68	−0.73	−0.55	−0.40	−0.85	−0.47	−0.43	− 0.49	− 0.60	− 0.55
TS2. Speed of traffic usually slow (30 mph)	0.34	0.34	0.41	0.38	0.44	0.34	0.32	0.58	0.63	0.47
TS3. Drivers drive faster than speed limit	−0.30	−0.48	−0.39	− 0.51	−0.39	− 0.58	−0.43	− 0.71	−0.58	− 0.37
Pedestrian infrastructure and safety (PI)										
PI1. Good lighting at night	0.36	0.43	0.47	0.60	0.37	0.50	0.55	0.48	0.36	0.64
PI2. Easy view of walkers / bikers from houses	0.39	0.41	0.36	0.41	0.43	0.68	0.40	0.38	0.65	0.59
PI3. Crosswalks and signals to cross busy streets	0.60	0.75	0.43	0.59	0.36	0.51	0.31	0.52	0.58	0.62
Safety from crime (CR)										
CR1. Fear of child being hurt by a stranger when alone outside around the home	−0.72	−0.89	−0.92	−0.91	−0.85	−0.78	−0.77	− 0.76	−0.84	− 0.66
CR2. Fear of child being hurt by a stranger when with a friend outside around the home	−0.73	− 0.91	−0.86	− 0.95	−0.96	− 0.90	−0.77	− 0.94	−0.92	− 0.57
CR3. Fear of child being hurt by a stranger when walking alone or with a friend in local streets	−0.98	− 0.95	−0.47	− 0.94	−0.77	− 0.87	−0.84	− 0.95	−0.97	− 0.94
CR4. Fear of child being hurt by a stranger when alone or with a friend in local park	−0.85	− 0.82	−0.32	− 0.91	−0.61	− 0.81	−0.72	− 0.87	−0.90	− 0.78
Aesthetics (AE)										
AE1. Interesting things to look at	0.90	0.77	0.76	0.63	0.76	0.78	0.70	0.54	0.70	0.78
AE2. Beautiful natural things to look at	0.88	0.59	0.66	0.83	0.82	0.74	0.78	0.73	0.65	0.85
AE3. Buildings / homes nice to look at	0.71	0.75	0.40	0.64	0.51	0.63	0.56	0.65	0.69	0.67
Average absolute inter-factor correlation	0.29	0.23	0.30	0.20	0.11	0.24	0.17	0.11	0.26	0.19
# of significant inter-factor correlations	8	7	8	6	4	6	5	2	9	5
Maximum absolute inter-factor correlation: pairs of factors	0.60	0.68	0.76	0.61	0.34	0.60	0.47	0.75	0.54	0.63
	TS-AE	AW-PI	AW-PI	AW-PI	AW-PI	TS-CR	TS-PI	PI-AE	TS-PI	AW-PI
Pairs of inter-correlated error items	3	3	1	1	2	4	0	0	0	1

*Notes.* NEWS-Y-IPEN = Neighborhood Environment Walkability Scale for Youth for the IPEN Adolescent study. ^a^New Zealand collected NEWS-Y-IPEN data from adolescents only. Thus, for New Zealand, confirmatory factor analyses were performed on adolescent data, while for the other countries they were performed using parents’ data

Based on results from the CFAs presented in this paper and extant NEWS-related algorithms developed for the IPEN Adult study [10], we devised a scoring protocol for the factor-analyzable and non-factor-analyzable subscales of the NEWS-Y-IPEN that optimizes cross-country comparability in the IPEN Adolescent study and other studies employing NEWS-Y-IPEN (see Additional file 2: Table S2). We provided single common (standard) scoring algorithms for all NEWS-Y-IPEN subscales with the exception of Residential density and Recreational facilities, for which two alternative algorithms were devised to account for differences in items across IPEN Adolescent countries (i.e., Denmark missing an item on the Residential density subscale; Nigeria missing three items on the Recreation facilities subscale).

### Between- and within-country variability in NEWS-Y-IPEN subscales

Table 4 shows the overall and country-specific descriptive statistics of scores on NEWS-Y-IPEN subscales. It also reports the proportion of total subscale variance attributable to between-country differences in scores. Residential density was the subscale with the highest level of between-country variability, followed by Safety from crime, Land use mix – diversity, and Aesthetics. For example, 42.2 and 29.9% of the total sample variances were attributable to between-country differences in scores on the Residential density and Safety from Crime subscales, respectively. Average perceived Residential density was highest in Hong Kong SAR and lowest in the USA (Baltimore, MD and Seattle, WA), while average perceived Land use mix – diversity was highest in Spain (Valencia) and lowest in Denmark (Odense). Both average perceived Safety from crime and Aesthetics were lowest among Bangladeshi (Dhaka) parents (Table 4). In the other subscales, the percentage of variance due to between-country differences was lower and ranged from 4.8 to 17.0%. The subscales showed sufficient levels of within-country variability, with most countries covering the full range of theoretical scores (1 to 5 for Land use mix diversity and Recreational facilities; 1 to 4 for the factor-analyzable subscales) on all subscales except for Residential density. Nevertheless, the within-country variability on the latter subscale was large (Table 4).

**Table 4:** NEWS-Y-IPEN subscales: descriptive statistics (means and standard deviations) and percentage of total subscale variance attributable to differences between countries

	NEWS-Y subscale (theoretical range of values)							
Country	Residential density (0–1000)	Land use mix – diversity (1–5)	Recreational facilities (1–5)	Accessibility & walking facilities (1–4)	Traffic safety (1–4)	Pedestrian infrastructure & safety (1–4)	Safety from crime (1–4)	Aesthetics (1–4)
Australia (Melbourne)	43.8 (83.8)	3.14 (0.81)	2.77 (0.88)	3.26 (0.48)	2.89 (0.53)	2.79 (0.54)	3.15 (0.76)	2.97 (0.76)
Bangladesh (Dhaka)	177.3 (84.7)	3.35 (0.61)	1.97 (0.73)	2.84 (0.57)	2.38 (0.58)	2.47 (0.60)	1.99 (0.87)	1.75 (0.72)
Belgium (Ghent)	69.2 (105.7)	3.52 (0.76)	2.77 (0.88)	2.98 (0.57)	2.50 (0.57)	2.67 (0.58)	3.11 (0.75)	2.27 (0.68)
Brazil (Curitiba)	95.4 (123.4)	3.14 (0.65)	2.45 (0.83)	2.91 (0.63)	2.15 (0.77)	2.56 (0.81)	2.01 (0.82)	2.37 (0.85)
Czech Republic (Olomouc; Hradec Králové)	131.4 (105.9)	3.28 (0.81)	3.00 (0.89)	3.17 (0.49)	2.87 (0.56)	3.01 (0.56)	2.85 (0.71)	2.24 (0.63)
Denmark (Odense)	103.9 (107.5)	2.29 (0.64)	2.62 (0.69)	3.07 (0.46)	2.94 (0.70)	2.92 (0.68)	3.67 (0.59)	2.66 (0.75)
Hong Kong SAR (Hong Kong)	468.7 (203.2)	3.47 (0.78)	2.88 (0.88)	2.99 (0.49)	2.82 (0.50)	2.95 (0.56)	2.69 (0.87)	2.47 (0.68)
India (Chennai)	65.9 (78.2)	3.41 (0.61)	1.78 (0.60)	2.58 (0.59)	2.27 (0.68)	2.93 (0.80)	3.02 (1.11)	1.51 (0.81)
Israel (Haifa)	220.3 (136.6)	3.07 (0.85)	2.47 (0.84)	3.13 (0.50)	2.31 (0.72)	2.85 (0.75)	3.13 (0.95)	2.39 (0.77)
Malaysia (Kuala Lumpur & other)	294.0 (230.0)	2.77 (0.78)	2.31 (0.88)	2.77 (0.43)	2.37 (0.53)	2.68 (0.58)	2.04 (0.71)	2.52 (0.63)
New Zealand^a^ (Auckland; Wellington)	57.5 (97.6)	2.86 (0.77)	2.69 (0.81)	2.88 (0.49)	2.90 (0.53)	2.81 (0.58)	3.67 (0.52)	2.72 (0.68)
Nigeria (Gombe)	269.4 (153.8)	3.43 (0.78)	3.08 (0.60)	2.73 (0.66)	2.96 (0.88)	2.97 (0.83)	2.73 (1.15)	2.93 (0.85)
Portugal (Various cities)	119.0 (91.7)	3.58 (0.74)	2.62 (0.93)	3.58 (0.42)	2.78 (0.45)	2.89 (0.49)	2.99 (0.53)	2.39 (0.52)
Spain (Valencia)	251.1 (134.7)	4.23 (0.50)	2.94 (0.80)	2.96 (0.36)	2.61 (0.72)	3.03 (0.62)	3.25 (0.79)	2.25 (0.74)
USA (Baltimore, MD; Seattle, WA)	31.1 (47.9)	2.81 (0.87)	2.87 (0.89)	2.90 (0.62)	2.58 (0.58)	2.83 (0.65)	3.01 (0.73)	3.08 (0.69)
All countries	205.1 (220.3)	3.24 (0.88)	2.71 (0.89)	2.98 (0.57)	2.65 (0.66)	2.85 (0.65)	2.87 (0.93)	2.53 (0.81)
% variance ADC	42.2	26.3	16.1	17.0	16.8	4.8	29.9	26.2

*Notes.* NEWS-Y-IPEN = Neighborhood Environment Walkability Scale for Youth for the IPEN Adolescent study; *ADC* attributable to differences between countries, *M* mean; *SD* standard deviation. ^a^New Zealand collected NEWS-Y-IPEN data from adolescents only. Thus, for New Zealand, analyses were performed on adolescent data, while for the other countries they were performed using parent-reported data

### Construct validity of NEWS-Y-IPEN

Table 5 reports covariate-adjusted pooled associations of binary objective measures of area-level SES and walkability with the NEWS-Y-IPEN subscales. As hypothesized above and in Table 5, scores on the Residential density, Land use mix – diversity, Recreational facilities, Accessibility and walking facilities, and Pedestrian infrastructure and safety subscales were positively related to area-level walkability. In line with our hypotheses, scores on the subscales of Recreational facilities, Traffic safety, Safety from crime, and Aesthetics were positively related to area-level SES. A positive (unexpected) association between Aesthetics and walkability was also observed. Findings did not significantly differ after excluding data from the few countries that used expert opinion to classify areas by SES and walkability (not presented).

**Table 5:** Associations of objectively-assessed binary measures of area-level socio-economic status (SES) and walkability with scores on the Neighborhood Environment Walkability Scale for Youth for the IPEN Adolescent study (NEWS-Y-IPEN)

NEWS-Y subscale	Hypotheses	Area-level SES (ref: low SES)		Area-level walkability (ref: low walkability)	
		*b* (95% CI)	*p*	*b* (95% CI)	*p*
Residential density	+ association with walkability	−3.45 (−11.96, 5.07)	.428	50.36 (41.77, 58.94)	<.001
Land use mix – diversity	+ association with walkability	0.01 (−0.03, 0.06)	.629	0.44 (0.39, 0.49)	<.001
Recreational facilities	+ association with SES and walkability	0.14 (0.08, 0.19)	<.001	0.16 (0.11, 0.21)	<.001
Accessibility & walking facilities	+ association with walkability	0.02 (−0.01, 0.05)	.215	0.24 (0.21, 0.28)	<.001
Traffic safety	+ association with SES	0.05 (0.02, 0.09)	.005	−0.01 (− 0.04, 0.03)	.576
Pedestrian infrastructure & safety	+ association with SES and walkability	0.00 (−0.04, 0.04)	.934	0.16 (0.12, 0.20)	<.001
Safety from crime	+ association with SES	0.16 (0.11, 0.21)	<.001	0.01 (−0.04, 0.06)	.635
Aesthetics	+ association with SES	0.18 (0.13, 0.22)	<.001	0.06 (0.02, 0.11)	.008

*Notes.* + = positive; *b* = regression coefficient point estimate, *CI* confidence intervals; ref. = reference category; *p* = *p*-value; all models adjusted for child’s age and sex, and country. Clustering at the neighborhood and/or school level accounted for

## Discussion

One of the main aims of the IPEN Adolescent project was to estimate pooled associations of perceived environmental attributes with physical activity and obesity using data from 15 countries across six continents. To achieve this aim, it was first necessary to harmonize the NEWS-Y-IPEN by establishing protocols producing summary scores that were comparable across countries. This had been previously performed for the IPEN Adult project [10]. In the IPEN Adult project, nearly all associations of physical activity and adiposity outcomes with the harmonized NEWS subscales were generalizable across countries [46–50]. Other international multi-country studies that have not developed harmonized scores for instruments measuring perceived neighborhood attributes found significant between-country differences in associations between perceptions of the environment and physical activity [51, 52]. Although these associations may vary by context, it is highly likely that the heterogeneous associations observed in those studies might have been due to methodological differences (e.g., between-country differences in measurement models or item interpretation).

At the NEWS-Y-IPEN item content / wording level, virtually no differences were found between IPEN Adolescent countries because all country-specific questionnaires were verified by the Coordinating Center prior to data collection. However, countries differed in the number of NEWS-Y-IPEN items included in their questionnaire (see Additional file 1: Table S1). Specifically, several countries included items that were not part of the original survey administered to the US sample of adolescents’ parents. These ‘additional’ items were particularly relevant for the country, but were omitted from this study because only items common to all main IPEN Adolescent study sites (including the USA) can be included in pooled analyses. The fact that Denmark excluded an item from the Residential density subscale capturing the presence of > 20-story residential buildings was not problematic because they were expecting 0 points on this item due to the lack of such buildings in the study site (Odense). Nigeria was the only country that included six rather than nine items in its Recreational facilities subscale, because investigators expected the three facility types (small and large public parks, school recreational facilities open to the public) would not be found in Gombe. To account for this difference, we have proposed two alternative scores for this subscale (Additional file 2: Table S2). Finally, New Zealand was the only country to administer the NEWS-Y-IPEN to adolescents rather than their parents.

CFAs of the a priori measurement model of the factor-analyzable items of the NEWS-Y-IPEN indicated that it did not provide a good fit to the data. As this was the first study to examine the factorial structure of NEWS-Y-IPEN, we are unable to compare our findings with those of prior studies. After excluding four items from the NEWS-Y-IPEN and re-specifying the structure of four latent factors, we derived well-fitting, comparable measurement models with five correlated latent factors: Accessibility and walking facilities, Traffic safety, Pedestrian infrastructure and safety, Safety from crime, and Aesthetics. Two of the omitted items (‘Parking is difficult in shopping areas’; ‘Presence of grass/dirt between streets and sidewalks’) were also omitted from the measurement models of NEWS used in the IPEN Adult project [10] and found to have low factor loadings in several other country-specific measurement models of the NEWS [14, 32, 36]. Another problematic item (‘Presence of trees along the street’) had substantially lower standardized loadings than other items measuring Aesthetics in the CFAs of NEWS for the IPEN Adult project [10] and the original version of NEWS [14]. Finally, the fourth omitted item (‘High crime rate’) was the only item of the Safety from crime subscale to refer to crime in general rather than specific criminal acts against a child. Hence, it is not surprising it did not show consistently high loadings on the latent factor it was supposed to measure.

Apart from the deletion of two items, the structures of the a priori latent factors of Safety from crime and Aesthetics did not require any re-specification. In contrast, re-specification was required for Pedestrian and automobile traffic safety, Land use mix – access, Walking / cycling facilities, and Street connectivity. The last three latent factors were merged into one factor in part due to the deletion of one of two Land use mix – access items and one of three Walking / cycling facilities items. The factor merging was also supported by the fact that previous CFAs of the NEWS for Adults indicated that Land use mix – access was strongly related to Street connectivity, whereby correlations ranging from 0.49 to 0.91 were observed in geographically-diverse IPEN Adult countries (Brazil, Mexico, New Zealand, Spain, and the UK) [10]). In the same study, high positive correlations (0.57 to 0.96) were found between Street connectivity and a factor encompassing items originally allocated to the a priori Walking / cycling facilities latent factor of the NEWS-Y-IPEN. The a priori latent factor of Pedestrian and automobile safety was split into two correlated latent factors: Traffic safety and Pedestrian infrastructure and safety (Table 3). The re-specified structure mirrored that of the NEWS for adults used in the IPEN Adult project [10]. Specifically, all NEWS-Y-IPEN items linked to the two re-specified factors consistently loaded on conceptually analogous factors in the NEWS for adults. In the present study, moderate-to-high positive inter-factor correlations were observed between Accessibility and walking facilities and Pedestrian infrastructure and safety in five of ten countries. Similar associations were observed between factors including comparable items in previous CFAs of NEWS for adults [10, 14, 32]. Overall, the above findings provide further support for the robustness and generalizability of the final factorial structure of the NEWS-Y-IPEN presented in this study.

One of the main reasons for conducting multi-country studies on the environment and physical activity is to increase variability in environmental exposures and health outcomes, which, in turn, makes it possible to more accurately estimate dose-response relationships [21]. Present analyses support this idea because 5 to 42% of the variability in NEWS-Y-IPEN subscale scores was attributable to differences between countries even after maximizing within-country variability in area-level walkability and SES by recruiting participants from selected communities. Two key components of walkability (residential density and land use mix) [20], Safety from crime, and Aesthetics displayed the highest levels of between-country variability. In general, samples located in cities with high population density (> 6500 persons/km^2^) and typified by high-rise residential buildings, such as Hong Kong (Hong Kong SAR) and Kuala Lumpur (Malaysia), had much higher perceived residential density than their counterparts [e.g., Melbourne (Australia), Auckland and Wellington (New Zealand), Seattle and Baltimore (USA)] [53]. Similar patterns were observed for the Land use mix – diversity subscale. On average, neighborhoods were perceived to be relatively safe from crime in most countries, except for Bangladesh (Dhaka), Brazil (Curitiba), and Malaysia (Kuala Lumpur and other cities) where parents reported being worried about letting their adolescent be outside the home unaccompanied by an adult. This finding is somewhat in line with international crime indices, according to which, Brazil, Bangladesh, and Malaysia ranked first, second, and fourth, respectively among the IPEN Adolescent countries [54]. Perceived aesthetics was generally higher in high income countries/regions [e.g., Australia (Melbourne), Hong Kong SAR (Hong Kong), and USA (Baltimore and Seattle)] and upper-middle-income countries [e.g., Brazil (Curitiba) and Malaysia (Kuala Lumpur and other cities)] than in lower-middle-income countries [Bangladesh (Dhaka) and India (Chennai)], with the exception of Nigeria (Gombe) where relatively high average scores were observed. Nigeria (Gombe) also displayed higher-than-expected scores for safety from crime, considering that it was the IPEN Adolescent country ranked third on an international crime index [54] and second on intentional homicide rate [55]. This might be due to cultural differences in the interpretation of the NEWS-Y-IPEN items, selection bias or contextual factors. Specifically, the Nigerian study was conducted in a “non-conflict city” of a conflict region [56, 57], so it is possible that parents in this city perceived higher neighborhood safety from crime relative to the other “conflict-states” in the north-eastern region of Nigeria. Also, it is generally and culturally acceptable in Northern Nigeria to have children play around in the neighborhoods without much concern about crime safety. Most of the violent crimes and terrorism tend to occur in crowded areas, such as places of worship, markets, schools, and government facilities.

In the IPEN Adult study, all subscales of NEWS for adults were found to be significantly related to at least one physical activity and obesity outcome [46–49]. The enhanced variability in exposures provided by pooling data from diverse countries also allowed the assessment and identification of curvilinear relations [48, 49]. It is yet to be seen whether pooled international NEWS-Y-IPEN data would yield similar associations in adolescents. Preliminary findings from single countries participating in the IPEN Adolescent project are suggestive of positive associations of adolescents’ PA with perceived Land use mix – diversity [13, 22, 58, 59], Aesthetics [13, 22, 59], Traffic safety [13, 22, 59], and Safety from crime [13, 22]. Divergent findings have been observed with respect to Residential density, Street connectivity, Pedestrian infrastructure and safety, and Recreational facilities [13, 22, 58–60]. By expanding the variability in perceptions of the neighborhood environment, the IPEN Adolescent project will allow a more robust estimation of these associations.

One of the aims of this study was to examine the construct validity of NEWS-Y-IPEN by estimating its associations with area-level SES and walkability. All hypothesized associations were confirmed. A dichotomous indicator of area-level walkability - operationalized as a composite index of dwelling density, street intersection density and land use mix [20] - was positively associated with perceived residential density, land use mix – diversity (proximity to services), accessibility and walking facilities, proximity to recreational facilities, and pedestrian infrastructure and safety. Previous studies using the NEWS for adults had also found positive associations of GIS-based area-level walkability with perceived residential density, proximity to services, and aspects of accessibility, pedestrian safety and infrastructure [36, 61]. In the present study, participants residing in high-SES neighborhoods reported higher scores on perceived proximity of recreational facilities, traffic safety, safety from crime, and aesthetics than their counterparts. Similarly, all these perceived attributes measured using a version of the NEWS for adults [36] and similar scales were found to be positively related to area-level household income [37, 38]. The present study extends the evidence of construct validity of the NEWS for adults to the NEWS-Y-IPEN, its version for youth.

### Limitations and strengths

Study limitations included the presence of a few between-country differences in neighborhood selection, recruitment strategies, survey administration, and sample sizes. We could not conduct CFAs on data from five of 15 IPEN Adolescent countries because their sample sizes were too small. Further, New Zealand administered the NEWS-Y-IPEN to adolescents rather than parents, and adolescents may interpret and respond to survey items differently than their parents [13]. Fortunately, the New Zealand measurement model of the NEWS-Y-IPEN fitted the data sufficiently well, indicating that the measurement models based on adolescent and parent responses may be similar. To shorten the NEWS-Y-IPEN and reduce attrition rates, the IPEN Adolescent coordinating center recommended omission of several destination items deemed less relevant to adolescents. As the relevance of these items was not examined in different countries, potentially important destinations for adolescents from various countries may have been omitted from the NEWS-Y-IPEN. Nigeria excluded several items measuring proximity to recreational facilities from their survey, which resulted in a restricted list of types of places to be included in the Recreational facilities subscale of the NEWS-Y-IPEN to be used in pooled analyses. The US sample omitted several items from their NEWS-Y-IPEN that were included in the original NEWS-Y. This reduced the number of available items measuring land use mix – access and street connectivity to one and two, respectively. As a result, the number of latent factors underlying the NEWS-Y-IPEN was also reduced (i.e., Land use mix – access and Street connectivity ended up being combined into one latent factor). Albeit a reduction in the number of items included in the NEWS-Y-IPEN may have some advantages for future studies because it lessens participant burden, it made it impossible for the present study to examine the importance of the omitted items for different populations of adolescents across the world. Future multi-site studies aiming to conduct pooled analyses should strive for greater measure and protocol fidelity to facilitate data pooling. As a result of the above-mentioned between-country differences in study protocol, we assessed between-country structural (*aka* configural) rather than full measurement-model equivalence of the NEWS-Y-IPEN, which is consistent with the NEWS for adults [10]. Specifically, we developed a common NEWS-Y-IPEN measurement model for all countries consisting of the same items and latent factors, which is necessary for the conduct of pooled analyses of the NEWS-Y-IPEN. Due to lack of relevant data, we could not test the retest-reliability of the NEWS-Y-IPEN across the participating sites. However, previously published data from the US and Hong Kong are suggestive of acceptable levels of repeatability [13, 16].

The variety of samples from countries with large differences in culture and environmental characteristics included in this study was a major strength. Other major strengths included use of comparable methods of participant recruitment and data collection across most participating study sites, stratified sampling strategy ensuring participants were balanced by two main environmental characteristics that impact PA (walkability and SES), and the contribution made to the assessment of both factorial and construct validity of the NEWS-Y-IPEN.

## Conclusions

We have derived a robust measurement model and common scoring protocol of NEWS-Y for the IPEN Adolescent project (NEWS-Y-IPEN) that, by improving inter-country comparability, will enhance the quality of pooled analyses of associations of the neighborhood environment with adolescents’ PA and health outcomes. Future studies employing NEWS-Y-IPEN should use the same scoring protocol to facilitate cross-study comparisons and interpretation of findings. Overall, the NEWS-Y-IPEN was found to possess good factorial as well as construct validity. A substantial percentage of the variability in NEWS-Y-IPEN summary scores was due to between-country differences, which is consistent with its adult counterpart [19, 46, 47]. This pattern suggests that the IPEN Adolescent project will be able to provide robust estimates of dose-response relationships between perceived attributes of the neighborhood environment, PA, and health outcomes of an international sample of adolescents.

## Supplementary information

**Additional file 1: Table S1.** Adaptation of the NEWS-Y for the IPEN Adolescent study (NEWS-Y-IPEN).

**Additional file 2: Table S2.** Country-specific scoring of the NEWS-Y-IPEN subscales for pooled analyses in the IPEN Adolescent study.

## Data Availability

The dataset supporting the conclusions of this article is available upon reasonable request to the international coordinating center of the IPEN Adolescent study.

## Abbreviations

CFA: Confirmatory factor analysis
CFI: Comparative fit index
GIS: Geographic Information Systems
IPEN: International physical activity and the environment network
NEWS: Neighborhood environment walkability scale
NEWS-Y: Neighborhood environment walkability scale for youth
NEWS-Y-IPEN: Neighborhood environment walkability scale for youth -
PA: Physical activity
RMSEA: Root mean square error of approximation
SES: Socio-economic status
SRMS: Standardized root mean square residual
