# Supplementary material for: Development and validation of the neighborhood environment walkability scale for youth across six continents
Source: Int J Behav Nutr Phys Act. 2019 Dec 3;16:122. doi: 10.1186/s12966-019-0890-6 (PMC6892154; doi:10.1186/s12966-019-0890-6)
Supplement: Supplementary file 1 — Additional file 1: Table S1. Adaptation of the NEWS-Y for the IPEN Adolescent study (NEWS-Y-IPEN). [file 12966_2019_890_MOESM1_ESM.docx]

**Table S1: Adaptation of the NEWS-Y for the IPEN Adolescent study (NEWS-Y-IPEN)**

| Original NEWS-Y subscale and items | Adaptations | Country-specific differences | Included in (CFA) *a priori*; final measurement models |
| --- | --- | --- | --- |
| ***Residential density*** | Original responses ranging from 1 to 5, with 1 = ‘none’. This results in positive density scores even in the absence of residential buildings. Responses were recoded as follows: 0 = none; 1 = a few; 2 = some; 3 = most; 4 = all. | None |  |
| 1. Separate or stand-alone one family homes | Wording changed to ‘Detached single-family residences’ | None | N/A; N/A |
| 1. Connected townhouses or row of houses | Wording changed to ‘Multi-family houses of 1-3 stories’. Density weight changed from 12 to 11 to correspond to the IPEN Adult version of the NEWS. | None | N/A; N/A |
| 1. Multiple-family or duplex homes | Wording changed to ‘Multi-family houses of 4-6 stories’. Density weight changed from 2 to 25 to reflect the average density of the modified item. | None | N/A; N/A |
| 1. Apartment or condo buildings | Wording changed to ‘Multi-family houses of 7-12 stories’. Density weight changed from 25 to 50 to reflect the average density of the modified item. | None | N/A; N/A |
|  | Added item ‘Multi-family houses of 13-20 stories’ with density weight 75. | None | N/A; N/A |
|  | Added item ‘Multi-family houses of over 20 stories’ with density weight 100. | Denmark did not include this item because the study site (Odense) does not have residential 20+ story residential buildings. | N/A; N/A |
| ***Land use mix - diversity*** | None to response scale. | None |  |
| 1. Convenience store/ corner store / small grocery store | None | None | N/A; N/A |
| 1. Supermarket | None | None | N/A; N/A |
| 1. Hardware store | Omitted to shorten the subscale. This type of destination is not considered very relevant to adolescents. | None | N/A; N/A |
| 1. Fruit / vegetable market | Omitted to shorten the subscale. This type of destination is not considered very relevant to adolescents. | None | N/A; N/A |
| 1. Laundry or dry cleaners | None | None | N/A; N/A |
| 1. Clothing store | Omitted to shorten the subscale. Probably not frequently visited destination by adolescents. | Several countries included this item in their survey. | N/A; N/A |
| 1. Post office | None | None | N/A; N/A |
| 1. Library | None | None | N/A; N/A |
| 1. Elementary school | Changed to “Any school”. | None | N/A; N/A |
| 1. Middle or high school | Changed to “Your child’s school” to assess distance to school as one of the main predictors of active transport to school. | None | N/A; N/A |
| 1. Bookstore | Omitted to shorten the subscale. Probably not frequently visited destination by adolescents. | Several countries included this item in their survey. | N/A; N/A |
| 1. Fast food restaurant | None | None | N/A; N/A |
| 1. Coffee place | None | None | N/A; N/A |
| 1. Bank / credit union | None | None | N/A; N/A |
| 1. Non-fast food restaurant | None | None | N/A; N/A |
| 1. Video store | Omitted to shorten the subscale. Probably not frequently visited destination by adolescents. | Several countries included this item in their survey. | N/A; N/A |
| 1. Pharmacy / drug store | None | None | N/A; N/A |
| 1. Hairdresser / barber shop | Omitted to shorten the subscale. Probably not frequently visited destination by adolescents. | None | N/A; N/A |
| 1. Any offices / worksites. | Omitted to shorten the subscale. This type of destination is not considered very relevant to adolescents. | None | N/A; N/A |
| 1. Bus, subway or train stop | None | None | N/A; N/A |
| ***Recreational facilities*** | None to the response scale. | None |  |
| 1. Indoor recreation or exercise facility (public or private) | None | None | N/A; N/A |
| 1. Beach, lake, river or creek | None | None | N/A; N/A |
| 1. Bike / hiking / walking trails, paths | None | None | N/A; N/A |
| 1. Basketball court | None | None | N/A; N/A |
| 1. Other playing fields / courts (e.g., soccer, skate park, etc.) | None | None | N/A; N/A |
| 1. YMCA | Omitted as not relevant in many countries. | None | N/A; N/A |
| 1. Boys and girls club | Omitted as more relevant to children than adolescents. | None | N/A; N/A |
| 1. Swimming pool | None | None | N/A; N/A |
| 1. Walking / running track | Omitted as covered by items 3 and 5. | None | N/A; N/A |
| 1. School with recreational facilities open to the public | None | Nigeria did not include this item. | N/A; N/A |
| 1. Small public park | None | Nigeria did not include this item. | N/A; N/A |
| 1. Large public park | None | Nigeria did not include this item. | N/A; N/A |
| 1. Public playground with equipment | Omitted as more relevant to children than adolescents. | None | N/A; N/A |
| 1. Public open space (grass or sand/dirt) that is not a park | Omitted to shorten the scale. | Several countries included this item in their survey. | N/A; N/A |
| ***Land use mix – access*** | None to the response scale. | None |  |
| 1. Stores within easy walking distance of our home | Omitted to shorten the scale. Attribute covered by the Land use mix – diversity scale. | Several countries included this item in their survey. | No; No |
| 1. Parking is difficult in shopping areas | None | None | Yes; No |
| 1. Many places for my child to go (alone/with someone) within easy walking distance | Omitted to shorten the scale. Attribute covered by the Land use mix – diversity scale. | Several countries included this item in their survey. | No; No |
| 1. Easy for my child to walk (alone/with someone) to a transit stop | Omitted to shorten the scale. Attribute covered by the Land use mix – diversity scale. | Several countries included this item in their survey. | No; No |
| 1. Hilly streets make it difficult for my child to walk in | None | None | Yes; Yes |
| 1. Major barriers to walking make it hard to get from place to place | Omitted to shorten the scale. | Several countries included this item in their survey. | No; No |
| ***Pedestrian and automobile traffic safety*** | None to the response scale. | None |  |
| 1. Difficult/unpleasant for my child to walk due to traffic in the neighborhood | None | None | Yes; Yes |
| 1. Speed of traffic usually slow (30 mph) | None | None | Yes; Yes |
| 1. Drivers drive faster than speed limit | None | None | Yes; Yes |
| 1. Good lighting at night | None | None | Yes; Yes |
| 1. Easy view of walkers / bikers from houses | None | None | Yes; Yes |
| 1. Crosswalks and signals to cross busy streets | None | None | Yes; Yes |
| 1. Lots of exhaust fumes while walking | Omitted to shorten the scale. Feature less important for pedestrian safety. | Several countries included this item in their survey. | No; No |
| ***Crime safety*** | None to the response scale. | None |  |
| 1. High crime rate | None | None | Yes; No |
| 1. High crime rate – unsafe to walk alone at night | Omitted to shorten the scale. Adolescents less likely to walk alone at night. | Several countries included this item in their survey. | No; No |
| 1. Fear of child being hurt by a stranger when alone outside around the home | None | None | Yes; Yes |
| 1. Fear of child being hurt by a stranger when with a friend outside around the home | None | None | Yes; Yes |
| 1. Fear of child being hurt by a stranger when walking alone or with a friend in local streets | None | None | Yes; Yes |
| 1. Fear of child being hurt by a stranger when alone or with a friend in local park | None | None | Yes; Yes |
| ***Aesthetics*** | None to the response scale. | None |  |
| 1. Presence of trees along the streets | None | None | Yes; No |
| 1. Presence of interesting things for my child to look at | None | None | Yes; Yes |
| 1. Presence of beautiful natural things for my child to look at | None | None | Yes; Yes |
| 1. Presence of buildings / homes nice for my child to look at | None | None | Yes; Yes |
| ***Walking /cycling facilities*** | None to the response scale. | None |  |
| 1. Presence of sidewalks on most of the streets | None | None | Yes; Yes |
| 1. Sidewalks separated from the road / traffic by parked cars | None | None | Yes; Yes |
| 1. Presence of grass / dirt between the streets and the sidewalks | None | None | Yes; No |
| ***Street connectivity*** | None to the response scale. | None |  |
| 1. Short distance between intersections | Omitted to shorten the scale. | Several countries included this item in their survey. | No; No |
| 1. Less cul-de-sacs in neighborhood | None | None | Yes; Yes |
| 1. Many different routes for getting from place to place in our neighborhood | None | None | Yes; Yes |
