# Supplementary material for: Development and validation of the neighborhood environment walkability scale for youth across six continents
Source: Int J Behav Nutr Phys Act. 2019 Dec 3;16:122. doi: 10.1186/s12966-019-0890-6 (PMC6892154; doi:10.1186/s12966-019-0890-6)
Supplement: Supplementary file 2 — Additional file 2: Table S2. Country-specific scoring of the NEWS-Y-IPEN subscales for pooled analyses in the IPEN Adolescent study. [file 12966_2019_890_MOESM2_ESM.docx]

**Table S2: Country-specific scoring of the NEWS-Y-IPEN subscales for pooled analyses in the IPEN Adolescent study**

| **Adapted NEWS-Y-IPEN subscale and items** | **Subscale version: Algorithm (item)** | **IPEN Adolescent country** |
| --- | --- | --- |
| ***Residential density*** |  |  |
| RD1. Detached single-family residences | Standard:  (RD1) + (RD2)*11 + (RD3)*25 + (RD4)*50 + (RD5)*75 + (RD6)*100  Alternative 1:  (RD1) + (RD2)*11 + (RD3)*25 + (RD4)*50 + (RD5)*75 | Australia, Bangladesh, Belgium, Brazil, Czech Republic, Hong Kong SAR, India, Israel, Malaysia, New Zealand, Nigeria, Portugal, Spain, USA  Denmark |
| RD2. Multi-family houses of 1-3 stories |  |  |
| RD3. Multi-family houses of 4-6 stories |  |  |
| RD4. Multi-family houses of 7-12 stories |  |  |
| RD5. Multi-family houses of 13-20 stories |  |  |
| RD6. Multi-family houses of over 20 stories |  |  |
| ***Land use mix – diversity*** |  |  |
| LD1. Convenience store/ corner store / small grocery store | Standard:  [(LD1) + … + (LD13)] / 13 | All |
| LD2. Supermarket |  |  |
| LD3. Laundry or dry cleaners |  |  |
| LD4. Library |  |  |
| LD5. Post office |  |  |
| LD6. Bank / credit union |  |  |
| LD7. Pharmacy / drug store |  |  |
| LD8. Any school |  |  |
| LD9. Your (child’s) school |  |  |
| LD10. Fast food restaurant |  |  |
| LD11. Coffee place |  |  |
| LD12. Non-fast food restaurant |  |  |
| LD13. Bus, subway or train stop |  |  |
| ***Recreational facilities*** |  |  |
| RF1. Indoor recreation or exercise facility (public or private) | Standard:  [(RF1) + … + (RF6)] / 6  Alternative 1:  [(RF1) + … + (RF9)] / 9 | All  Australia, Bangladesh, Belgium, Brazil, Czech Republic, Denmark, Hong Kong SAR, India, Israel, Malaysia, New Zealand, Portugal, Spain, USA |
| RF2. Beach, lake, river or creek |  |  |
| RF3. Bike / hiking / walking trails, paths |  |  |
| RF4. Basketball court |  |  |
| RF5. Other playing fields / courts (e.g., soccer, skate park, etc.) |  |  |
| RF6. Swimming pool |  |  |
| RF7. School with recreational facilities open to the public |  |  |
| RF8. Small public park |  |  |
| RF9. Large public park |  |  |
| ***Accessibility and walking facilities*** |  |  |
| AW1. Hilly streets make it difficult to walk in the neighborhood | Standard:  [(AW1_R) + (AW2) + (AW3) + (AW4) + (AW5)] / 5 | All |
| AW2. Less cul-de-sacs in the neighborhood |  |  |
| AW3. Many different routes for getting from place to place in our neighborhood |  |  |
| AW4. Presence of sidewalks on most of the streets |  |  |
| AW5. Sidewalks separated from the road / traffic by parked cars |  |  |
| ***Traffic safety*** |  |  |
| TS1. Difficult/unpleasant to walk due to traffic in the neighborhood | Standard:  [(TS1_R) + (TS2) + (TS3_R)] / 3 | All |
| TS2. Speed of traffic usually slow (30 mph) |  |  |
| TS3. Drivers drive faster than speed limit |  |  |
| ***Pedestrian infrastructure and safety*** |  |  |
| PI1. Good lighting at night | Standard:  [(PI1) + (PI2) + (PI3)] / 3 | All |
| PI2. Easy view of walkers / bikers from houses |  |  |
| PI3. Crosswalks and signals to cross busy streets |  |  |
| ***Safety from crime*** |  |  |
| CR1. Fear of child being hurt by a stranger when alone outside around the home | Standard:  [(CR1_R) + (CR2_R) + (CR3_R) + (CR4_R] / 4 | All |
| CR2. Fear of child being hurt by a stranger when with a friend outside around the home |  |  |
| CR3. Fear of child being hurt by a stranger when walking alone or with a friend in local streets |  |  |
| CR4. Fear of child being hurt by a stranger when alone or with a friend in local park |  |  |
| ***Aesthetics*** |  |  |
| AE1. Interesting things to look at | Standard:  [(AE1) + (AE2) + (AE3)] / 3 | All |
| AE2. Beautiful natural things to look at |  |  |
| AE3. Buildings / homes nice to look at |  |  |

*Notes.* NEWS-Y-IPEN = Neighborhood Environment Walkability Scale for Youth for the IPEN Adolescent study; _R = reverse scored item.
